# The impact of socioeconomic status and lifestyle on cognitive aging and brain health: results from the LIFE-Adult-Study

**DOI:** 10.1186/s13195-026-02017-4

**Published:** 2026-03-24

**Authors:** Andrea E. Zülke, Melanie Luppa, Laurenz Lammer, Susanne Röhr, Ronny Baber, Ronald Biemann, Kerstin Wirkner, Samira Zeynalova, Maryam Yahiaoui-Doktor, Silke Zachariae, Christoph Engel, Andreas Hinz, Heide Glaesmer, Matthias L. Schroeter, Markus Löffler, Arno Villringer, A. Veronica Witte, Frauke Beyer, Steffi G. Riedel-Heller

**Affiliations:** 1 Institute of Social Medicine, Occupational Health and Public Health, Leipzig University, Leipzig, Germany; 2 Department of Neurology, Max Planck Institute for Human Cognitive and Brain Sciences, Leipzig, Germany; 3 Global Brain Health Institute (GBHI), Trinity College Dublin, Dublin, Ireland; 4 Centre for Healthy Brain Ageing (CHeBA), School of Clinical Medicine, UNSW Sydney, Sydney, Australia; 5 Institute of Laboratory Medicine, Clinical Chemistry and Molecular Diagnostics, University Hospital Leipzig, Leipzig, Germany; 6 Leipzig Research Centre for Civilization Diseases, Leipzig University, Leipzig, Germany; 7 Institute for Medical Informatics, Statistics and Epidemiology, Leipzig University, Leipzig, Germany; 8 Department of Medical Psychology and Medical Sociology, Leipzig University, Leipzig, Germany; 9 Cognitive Neurology, University of Leipzig Medical Center, Leipzig, Germany; 10 Bordeaux Population Health Research Center, University of Bordeaux, Inserm, UMR 1219, Bordeaux, France

**Keywords:** Lifestyle, Health inequalities, Dementia, Risk factors, Cognitive function, Prevention

## Abstract

**Background:**

Dementia risk and changes in cognitive performance follow a social gradient, however, evidence on the respective role of socioeconomic status (SES) and lifestyle for cognitive performance is inconclusive. We investigated effects of SES and lifestyle (Lifestyle for Brain Health (LIBRA)-index) on cognitive performance and neuroimaging markers.

**Methods:**

We analyzed data from the registry-based LIFE-Adult-Study (*n* = 1,581; M_age_ = 63.6, SD = 10.1; 45.9% female). Multivariable regression models examined associations of SES and LIBRA with cognitive performance and MRI-derived hippocampal volume and white matter hyperintensities (WMH) at follow up, as well as changes in LIBRA from baseline to follow-up, controlling for relevant covariates and time between baseline and follow-up assessment (mean: 6.6 years).

**Results:**

Higher SES was associated with better cognitive performance at follow-up (b = 0.12, 95% CI: 0.01, 0.24). No linear association was found between LIBRA and cognition, but higher squared LIBRA-scores predicted lower cognitive performance (b = –0.007, 95% CI: –0.01, –0.001). No significant interactions between SES and LIBRA were observed when investigating changes in cognitive performance. LIBRA scores worsened more strongly among participants with low SES (b = 0.12, 95% CI: 0.03, 0.20). Detrimental changes in LIBRA were linked to higher WMH volume at follow-up (b = 0.02, 95% CI: 0.003; 0.03), while no effects on hippocampal volume were detected.

**Discussion:**

Low SES predicted negative change in cognitive performance, while lifestyle changes showed an effect only beyond a certain threshold. Although no interaction between SES and lifestyle was found when predicting changes in cognitive performance, higher LIBRA-scores among low-SES individuals highlights the need for targeted prevention. Effects of LIBRA on WMH volume supports white matter as a potential early marker of lifestyle-related brain health.

**Supplementary Information:**

The online version contains supplementary material available at 10.1186/s13195-026-02017-4.

## Background

It was estimated that 57 million people were living with dementia globally in 2019. This figure is expected to increase to 153 million by 2050, particularly due to population ageing and population growth observed in most countries [1]. In Germany, 1.8 million people are currently living with dementia [2], with actual numbers expected to be even higher due to low detection rates in primary care [3, 4]. The age-adjusted incidence of dementia has been declining by 13% per decade in Europe and the United States, which has, in part, been attributed to changing prevalences of modifiable risk factors [5, 6]. In the absence of curative and effective treatment options, research on modifiable risk and protective factors for dementia has been accumulating rapidly in recent years, highlighting the potential for risk reduction by risk factor modification, as evidenced e.g. in the reports of the Lancet Commission on Dementia Prevention, Intervention and Care [7]. Well-established risk factors include, for example, hypertension, diabetes mellitus, physical inactivity, smoking or depression, while evidence is accumulating for further individual-level (e.g., anxiety and post-traumatic stress disorder, leisure activities, purpose in life) and population-level (e.g., exposure to greenness, area deprivation) risk- and protective factors [8].

Incidence of dementia follows a socioeconomic gradient, disproportionately affecting people with lower socioeconomic status (SES; [9–11]). In-depth investigations of the respective contributions of lifestyle and socioeconomic factors (commonly comprising income, education and occupational status) on cognitive decline and dementia risk are crucial to inform appropriate strategies for dementia risk reduction and healthy brain ageing. To date, most (national) strategies towards dementia risk reduction focus on individual behavior change, i.e., educating adults on the links between lifestyle-based risk factors and promoting healthy behavior changes on an individual level. However, this approach is deemed insufficient to reduce dementia cases on a population level and might likely increase health inequalities [12]: First of all, many risk factors for dementia are highly prevalent, requiring distribution of resources to large numbers of people. Beyond that, exposure to risk factors is distributed unequally across societies, e.g., socioeconomically disadvantaged individuals tend to live in areas less favorable for physical activity and social interactions and more often work in occupations with exposure to loud noise or shift work, which implies structural barriers to engaging in a healthy lifestyle. Population-level approaches are deemed a promising approach to reduce risk exposure for whole parts of societies, e.g. by measures like smoking bans or the expansion of bike lanes, which actively shape environments and rely less on conscious behavior change.

Evidence regarding the respective contributions of SES and modifiable (lifestyle) factors for cognitive performance and dementia risk has been inconclusive. While a population-based study from the Netherlands found 52% of risk differences between SES-strata to be due to differences in a validated dementia risk score, comprising 12 modifiable risk factors (Lifestyle for Brain health (LIBRA)-score; [13]), other studies estimated proportions of SES-differences mediated by lifestyle between 3.2 and 12% [14–16]. Some studies reported stronger associations of lifestyle factors and cognitive performance in low-SES groups [17–19], while others found stronger correlations between lifestyle and risk for all-cause dementia and Alzheimer’s disease (AD) particularly among high-SES individuals [20], or found no SES-lifestyle-interaction when predicting incident dementia cases [15].

Certain studies also assessed associations between a brain-healthy lifestyle and structural brain markers. A Chinese study reported that individuals in the highest tertile of LIBRA-scores had increased odds of intracranial atherosclerotic plaque and intracranial atherosclerotic burden [21]. Bransby and colleagues [22] report that multiple dementia risk factors (compared to no/single risk factors) were linked to p-tau 181-accumulation, cerebrospinal fluid (CSF)-tau and worse cognition, but not Aβ or brain volume cross-sectionally in cognitively unimpaired middle-aged and older Australians. Rosenich et al. [23] found a lower prevalence of modifiable risk factors to be linked to less hippocampal volume (HCV) loss in Aβ-negative, cognitively healthy older Australians. In the US POINTER-trial, testing a multidomain lifestyle intervention against cognitive decline, cardiovascular risk factors (Framingham Risk Score) were cross-sectionally linked to lower HCV and cerebral grey matter (GM) volumes, as well as higher white matter hyperintensity (WMH) volume, but not Aβ- or tau-burden [24]. In the population-based Maastricht study, higher LIBRA-scores (higher dementia risk) were linked to higher WMH in men only. CSF and WMH partly mediated associations of LIBRA with cognitive performance [25]. Using a custom healthy lifestyle score (healthy diet, physical activity, non-smoking, moderate alcohol consumption, healthy body weight), Pan and colleagues [26] found longitudinal associations between a healthy lifestyle and larger total brain volume, GM volume, white matter volume, HCV and WMH in participants of the UK Biobank. Further based on UK Biobank data, Ou et al. [20] reported that the combination of higher SES and healthier lifestyle was associated with significantly higher cortical and subcortical volumes. A British birth cohort study found longitudinal associations between higher Framingham Risk Scores and smaller brain volumes, as well as greater WMH. Associations were strongest for risk factors prevalent in early adulthood [27]. In middle-aged and older participants from a US-based Alzheimer’s prevention registry, LIBRA was not associated with Aβ-accumulation or longitudinal cognitive trajectories [28].

Respective studies from Germany or other European countries are currently scarce, highlighting the need for further investigation of longitudinal associations between the LIBRA-index and structural brain health markers. Since AD-related pathophysiological processes begin decades before the manifestation of clinical symptoms [29–31], studies assessing samples of younger, e.g., middle-aged adults seem particularly relevant. Against this background, the present study investigates associations between SES, modifiable risk factors for dementia, assessed using the LIBRA-score, and changes in cognitive performance, using a registry-based sample of middle-aged and older German adults. Further, we assess whether modifiable risk factors for dementia correlate with (changes in) structural brain markers, and whether SES and further covariates explained changes in risk factors for dementia in secondary analyses.

## Material and methods

### Study design and participants

This study uses data from the population-based LIFE-Adult-Study. LIFE-Adult comprises an age- and sex-stratified random sample of 10,000 inhabitants of the city of Leipzig, Germany at baseline. Participants were randomly sampled from the residents’ registration office of the city of Leipzig and invited by mail, including an information leaflet and response form; non-responders received a reminder letter and were subsequently contacted by phone where possible. The overall response rate was 31% among individuals aged 40–79 years [32]. LIFE-Adult aims to assess the prevalence and incidence of civilization diseases (e.g., depression, dementia, stroke) and investigate interactions between genetic and lifestyle factors in the occurrence of said conditions. Eligible participants were aged between 18 and 79 years at baseline, with a focus on adults aged 40 to 79 years. Pregnancy and insufficient command of the German language were exclusion criteria. Pregnant women were invited to participate in the LIFE-Child-Study, a longitudinal population-based childhood cohort study [33]. Comparisons with official population statistics indicated that participants were more often married, employed, healthier, and less likely to smoke than the general Leipzig population and non-participants. Socioeconomic differences between the study sample and the city of Leipzig were particularly pronounced for men, i.e., among male study participants aged 50 to 69, the frequency of a high education level was 1.5 times higher than in the respective share of the overall population of Leipzig, indicating an overrepresentation of individuals with higher SES compared to the city population [34].

Baseline assessments were conducted between 08/2011 and 11/2014 and included physical examinations, computer-assisted personal interviews, psychometric tests, self-administered questionnaires and clinical chemistry based on blood- and urine samples. An additional brain MRI examination including cognitive performance tests was conducted in a subsample of participants aged 40 to 79 years (~ n = 2,600). The follow-up assessment (10/2017–08/2021) included two parts: First, all LIFE-Adult participants received a set of paper-based questionnaires to complete at home. Participants of the MRI-examination at baseline were invited for an additional visit to the study center for physical examinations, interviews, psychometric tests and self-administered questionnaires, as well as a second MRI-assessment. A total of 5,512 and 1,799 participants completed the written and physical follow-up examination, respectively. Further details on the LIFE-Adult-Study and assessments conducted at baseline and (written and physical) follow-up can be found elsewhere [32, 35].

### Outcomes and covariates

#### Lifestyle-based risk factors for dementia

Modifiable risk factors for dementia were operationalized using the LIBRA-index. LIBRA is a validated score which has been shown to predict cognitive decline and dementia in several population-based cohorts of middle-aged and older adults [13, 36–38]. The score comprises 12 modifiable lifestyle- and health factors, identified through a systematic literature review and Delphi consensus study, i.e.: diabetes mellitus, obesity, hypertension, chronic kidney disease, heart disease, hypercholesterolemia, physical inactivity, diet, high alcohol consumption, smoking, depression, and cognitive activity. Each factor is assigned a weight (positive/negative for risk/protective factors, respectively) based on the relative risk of said factor [39]. Weights are summed up to calculate the overall LIBRA-score, ranging from −5.9 to + 12.7 points, with higher scores indicating higher risk for dementia or more room for improvement. All LIBRA-factors except for high cognitive activity (assessed based on occupation-related cognitive abilities, using LIFE-baseline-data) were assessed in both baseline and follow-up examinations. Therefore, we calculated the LIBRA-score based on 11 risk factors, scores ranging from −2.7 to 12.7 points. Operationalization of all LIBRA-factors in the LIFE-Adult-Study is described in Supplementary Table 1.

#### Cognitive performance

We assessed cognitive performance using a composite z-score, based on selected cognitive tests from the Consortium to Establish a Registry for Alzheimer’s Disease (CERAD)-Plus neuropsychological test battery [40, 41]. Assessments included the Trail Making Test A and B (TMT-A, TMT-B) for task switching and processing speed, and the Verbal Fluency Test (VFT) “animals” as a measure of semantic fluency and semantic memory. TMT-A requires participants to connect consecutive numbers as quickly as possible, while in the TMT-B, consecutive numbers and letters are to be connected alternatingly. Time (in seconds) to complete the tasks constitute the test scores, with lower values indicating better performance. For the VFT, participants were instructed to name as many animals as possible in 60 s. The number of correctly given animal names constitute the VFT-score, higher scores indicating better test performance. Scores of the TMT-A and TMT-B were inverted in a first step, so that higher scores indicated better cognitive performance in all cognitive tests. Individual test scores were then z-standardized and summed up, with the mean of the derived z-score constituting the composite measure of cognitive performance.

#### Socioeconomic status

Socioeconomic status (SES) was assessed based on education, occupational status and net equivalent income at baseline, following established procedures [42]. For *education*, the SES-index uses information on school and vocational education, categorized into low, middle and high levels following the Comparative Analysis of Social Mobility in Industrial Nations (CASMIN)-classification [43]. *Income* included income from paid work, capital, pensions and other sources of income of all household members, contributing to the needs-weighted net equivalent income, taking into account the number of household members. *Occupational status* was classified into lower and higher categories based on the International Socio-Economic-Index of Occupational Status (ISEI). If a household consisted of more than one person, the highest occupational status of the respective household was selected. All three domains (education, income, occupational status) had a value between 1 and 7 and contributed equally to the total SES-score (range: 3–21), whereas higher scores indicate higher SES. Participants were assigned a low, middle and high SES based on sample distributions.

#### Covariates

Participants provided information on their age and sex (binary, male/female) during the baseline examination. We further included relationship status (married or living in a relationship vs. single, divorced or widowed), employment status (employed vs. retired vs. unemployed/being a homemaker), and living situation (living alone vs. living together with others) based on participants’ self-report in our analyses. Further covariates included social isolation, assessed using the 6-item version of the Lubben Social Network Scale (LSNS-6; [44]). The LSNS-6 assesses numbers of and frequency of contacts with family and friends, with each item receiving a value between 0 and 5 points. A total sum score (range: 0–30 points) is created, with scores ≤ 11 indicating risk of social isolation. We included perceived social support, based on the 5-item version of the ENRICHD social support inventory (ESSI). The ESSI captures aspects of social support (instrumental, emotional, appraisal, informational) using a 5-point Likert-scale (item scores: 1–5 points), with sum scores ranging from 5 to 25 and higher scores indicating higher levels of social support [45]. Since time intervals between baseline- and follow-up examination varied across the sample (range: 4.2–9.3 years; mean: 6.6), an indicator of time between the two assessments was included in all regression analyses. Lastly, to control for baseline differences in LIBRA-scores, cognitive performance and MRI-markers, respectively, and to counteract the risk of regression to the mean [46, 47], baseline-values for LIBRA, cognitive performance and imaging markers were included in analyses as covariates, respectively.

#### Brain imaging markers

A subsample of LIFE Adult (n ~ 2.600) participated in magnetic resonance imaging (MRI)-examinations at baseline. Structural brain imaging was conducted using a 3 Tesla Siemens Verio MRI scanner (Siemens Healthcare, Erlangen, Germany) equipped with a 32-channel head coil. T1-weighted images were acquired using a 3D MPRAGE sequence based on the ADNI standard protocol, with the following parameters: inversion time = 900 ms, repetition time = 2300 ms, echo time = 2.98 ms, flip angle = 9°, field of view = 256 × 240 × 176 mm^3^, matrix size = 256 × 240, voxel size = 1 × 1 × 1 mm^3^, no interpolation, no fat suppression, and whole-brain coverage with tune-up shimming. Images were acquired in sagittal orientation.

Assessment of WMH in the LIFE-Adult-Study is described in detail elsewhere [48]. WMH were segmented using the longitudinal pipeline of the Lesion Segmentation Toolbox (version 3.0.0, run on MATLAB version 9.10; [49]) This pipeline estimates the location of stable lesions as well as regression and progression of lesions over time [50]. First, we performed cross-sectional lesion segmentation using the Lesion Prediction algorithm with its default parameters. Then, we applied the longitudinal pipeline to the cross-sectional runs and obtained voxel-wise maps of lesion change (LCL maps). In these three-valued whole-brain maps, 1 indicates a regression of lesion volume, 2 indicates a stable lesion, and 3 indicates a newly appeared lesion in this voxel. For baseline lesion volume, we summed up the volume of all LCL voxels with a value of 2 and for follow-up lesion volume, we added the volumes of all LCL voxels with a value of 1 (regressed lesion voxels) or 3 (novel lesion voxels). Participants with major artifacts or incidental findings were excluded.

To assess HCV, we processed the MRIs with FreeSurfer version 7.4.1, employing its longitudinal sequence-adaptive multimodal segmentation (SAMSEG; [51]) to derive hippocampal and total intracranial volume [52].

We averaged HCV over both hemispheres and adjusted it for intracranial volume (ICV) according to the following formula:

$$\mathrm{HCV}\:=\:\mathrm{HCVraw}-\beta\:\ast\:(\mathrm{ICVraw}\:-\:\mathrm{ICVmean}),$$

where β is the unstandardized regression coefficient of HCV on intracranial volume (ICV) from a linear mixed-effects model and i denotes the observation. ICV was added as a control variable to models with WMH as a dependent variable.

### Statistical analyses

Missing values were imputed using multiple imputation by chained equations. To evaluate the missing at random (MAR) assumption, we examined whether missingness in any of the *n* = 11 LIBRA-components, covariates, and cognitive tests was associated with observed participant characteristics. Missing values in several lifestyle-related variables were associated with age, sex, and SES, whereas missingness in cognitive performance was not linked to age, sex, or SES (see Supplementary Table 2). As missingness was systematically related to observed variables that were included in the imputation model, but not to the cognitive outcome itself, we considered the MAR assumption to be plausible, and assumed no critical violation of the MAR-assumption. Variables with incomplete data were imputed one at a time, applying regression models conditional on all other variables in the imputation model. We applied predictive mean matching for continuous variables (k = 10 nearest neighbors), ordinal logistic regression for ordinal variables and logistic regression with augmentation for binary variables. All variables used in the analysis were included in the imputation model. The imputation model generated 20 imputed datasets with 500 iterations per dataset, following a burn-in of three iterations for stability. A random seed was applied to secure reproducibility. The variables age, sex, and SES were registered as regular predictors to guide the imputation process. Imputations were performed at the item level of the LIBRA, and the total LIBRA-score was calculated following imputation. MRI-derived imaging data were not imputed. All regression analyses used the pooled results of 20 imputed datasets, with pooled estimates obtained according to Rubin’s rules as implemented in the *mi estimate*-command of Stata 16. Convergence of imputation models was assessed by visual inspection of iteration plots for selected variables, with no indication of trends.

Sample characteristics are described using means (standard deviations) or percentages, as appropriate. Group differences, e.g., according to SES, were assessed using χ^2^ tests or ANOVA, as appropriate. As values for LIBRA (total score) and WMH showed slightly skewed distributions, respective values were square-root (LIBRA) and log-transformed (WMH) prior to regression analyses. Multivariable linear regression models were used to investigate the following outcomes:

1. Cognitive performance at follow-up (composite z-score) as a function of baseline cognitive performance, lifestyle (change in LIBRA score from baseline to follow-up, ΔLIBRA and ΔLIBRA^2^), and SES (baseline):

Cognitive performance (follow-up) = β_0_ + β_1_*age + β_2_*sex + β_3_*SES + β_4_*cognitive performance (baseline)+ β_5_*ΔLIBRA + β_6_*(ΔLIBRA)^2^+ β_7_*living situation + β_8_*partnership + β_9_*employment status + β_10_*social support + β_11_*social isolation+ β_12_*time between baseline and follow-up + ε;

2. Lifestyle at follow-up (LIBRA score) as a function of baseline SES,:

LIBRA (follow-up) = β_0_ + β_1_*age + β_2_*sex+ β_3_*SES + β_4_*cognitive performance (baseline) + β_5_*LIBRA (baseline)+ β_6_*living situation + β_7_*partnership+ β_8_*employment status + β_9_*social support + β_10_*social isolation+ β_11_*time between baseline and follow-up + ε;

3. Neuroimaging markers at follow-up (HCV, WMH volume) as a function of lifestyle (change in LIBRA score from baseline to follow-up, ΔLIBRA and ΔLIBRA²) and SES (baseline).

HCV (follow-up) = β_0_ + β_1_*age + β_2_*sex+ β_3_*SES + β_4_*cognitive performance (baseline) + β_5_*ΔLIBRA+ β_6_*(ΔLIBRA)^2^+ β_7_*living situation + β_8_*partnership+ β_9_*employment status + β_10_*social support + β_11_*social isolation+ β_12_*time between baseline and follow-up + β_13_*HCV (baseline) + ε;

WMH volume (follow-up) = β_0_ + β_1_*age + β_2_*sex+ β_3_*SES + β_4_*cognitive performance (baseline) + β_5_*ΔLIBRA+ β_6_*(ΔLIBRA)^2^+ β_7_*living situation + β_8_*partnership + β_9_*employment status + β_10_*social support + β_11_*social isolation+ β_12_*time between baseline and follow-up + β_13_*WMH volume (baseline) + ε.

All models were adjusted for age, sex, education, employment status, living situation, partnership status, social isolation, social support (at baseline, respectively), baseline-values of respective outcomes and time between baseline and follow-up. Analyses were based on pooled results from 20 multiply imputed datasets. Linear and quadratic effects of change in LIBRA were explored to investigate potential non-linear or threshold effects of lifestyle changes on cognitive performance and neuroimaging markers, following the approach of Heger and colleagues [25]. Model fit of regression models for the primary outcome cognitive performance with and without the quadratic term (ΔLIBRA^2^) were conducted using the Akaike Information criterion, favoring the model including ΔLIBRA^2^ (3124.24 vs. 3127.68; not tabulated). An alpha-level of 0.05 (two-tailed) was applied in all tests. The primary endpoint was cognitive performance at follow-up. Secondary endpoints comprised lifestyle (LIBRA) at follow-up and neuroimaging markers (WMH, HCV). To account for multiple testing in secondary analyses, *p*-values were adjusted using the Benjamini–Hochberg procedure to correct the false discovery rate (FDR). Families of tests were defined a priori as the sets of theoretically relevant predictors within each secondary outcome model (i.e., SES and psychosocial variables in the model describing LIBRA-scores at follow-up; ΔLIBRA and ΔLIBRA^2^ in the models describing WMH and HCV at follow-up). Unless otherwise stated, results refer to adjusted *p*-values, unadjusted values reported alongside for transparency. Variance inflation factors (VIFs) were calculated to rule out major sources of collinearity. Analyses were conducted using Stata (SE) 16.0.

## Results

### Descriptive analyses

We excluded all participants who did not take part in the physical follow-up examination, which included the cognitive assessments (*n* = 8,196), or who were younger than 40 years at baseline (*n* = 217), as the LIBRA-index was particularly designed to measure dementia risk in midlife. After excluding participants with missing values on SES (*n* = 6), the final sample contained *n* = 1,581 individuals. Among participants with a migration background (5.6%), the most common countries of origin were Poland (53%), the Czech Republic/Czechoslovakia (13%), and Hungary (8%), with other countries each accounting for less than 3%. Usable MRI-data for HCV and WMH were available for *n* = 902 and *n* = 889 participants, respectively. Descriptive characteristics of participants at baseline are provided in Table 1 (unimputed data). Baseline characteristics of participants stratified by SES are provided in Supplementary Table 3.

**Table 1:** Characteristics of participants at baseline (*n* = 1,581)

Variable	n (%)	Missing values, n (%)
Age, Mean (SD); range	63.6 (10.1); 40.1–80.3	
Sex		
women	726 (45.9)	
men	855 (54.1)	
*Socioeconomic status*		
low	245 (15.5)	
intermediate	955 (60.4)	
high	381 (24.1)	
*Employment status*		4 (0.003)
employed	619 (39.3)	
retired	913 (57.9)	
unemployed, homemaker	45 (2.9)	
*Country of birth*		
Germany	1,493 (94.4)	
other	88 (5.6)	
*Living situation*		2 (0.001)
living alone	312 (19.8)	
living together with others	1,267 (80.2)	
*Relationship status*		
in a relationship	1,284 (81.2)	
single, divorced, widowed	297 (18.8)	
*Social isolation*		175 (11.1)
socially isolated (LSNS < 12)	197 (14.0)	
socially integrated (LSNS ≥ 12)	1,209 (86.0)	
Perceived social support (ESSI), Mean (SD)	22.3 (3.5)	34 (2.2)
LIBRA-Score (total), Mean (SD)	1.5 (2.1)	386 (24.4)^#^
Cognitive performance (z-score), Mean (SD)	0 (0.9)	14 (1.0)
Hippocampal volume (1,000m^3^), Mean (SD)	8.3 (0.9)	679 (42.9)
Volume of right hippocampus (1,000m^3^), Mean (SD)	4.2 (0.4)	679 (42.9)
Volume of left hippocampus (1,000m^3^), Mean (SD)	4.1 (0.4)	679 (42.9)
White matter hyperintensity volume (1,000m^3^), Mean (SD)	2.18 (5.24)	692 (43.8)
Total intracranial volume (1,000m^3^), Mean (SD)	1,572.0 (142.4)	679 (42.9)

*ESSI* ENRICHD social support inventory, *LIBRA* Lifestyle for Brain Health Index, *LSNS* Lubben Social Network Scale, *ref*. reference, *SD* Standard deviation

^#^: coded “missing” if ≥ 1 component of LIBRA was missing

Older participants had more missing values on alcohol consumption (*p* = 0.0002), social support (*p* = 0.037), social isolation (*p* = 0.002), physical activity (*p* < 0.001), healthy diet (*p* = 0.0004) and smoking (*p* = 0.0007). In women, more values were missing on social support (*p* = 0.010), hypertension (*p* = 0.016), physical activity (*p* = 0.002), and smoking (*p* = 0.025), while information on social isolation was more often missing for men (*p* < 0.001). Participants with a low SES more often had missing values on alcohol consumption (*p* < 0.0001), social support (*p* = 0.019), hypertension (*p* = 0.040), physical activity (*p* < 0.0001), healthy diet (*p* = 0.034), and smoking (*p* < 0.0001). An intermediate SES was linked to missing values for alcohol consumption (*p* = 0.028), physical activity (*p* < 0.0001), and smoking (*p* = 0.018). Missing values in cognitive performance were not linked to age, sex, or SES. Associations of missing values in all imputed variables with age, sex and SES are described in detail in Supplementary Table 2.

Regarding individual LIBRA-factors at baseline, differences between SES-groups were detected for diabetes (*p* = 0.048), depression (*p* = < 0.001), smoking (*p* = 0.037), obesity (*p* = 0.007), hypertension (*p* = 0.007), alcohol consumption (*p* = < 0.001), with respective risk factors being more common among the low-SES group. No SES-differences were found for a healthy diet (*p* = 0.787), renal disease (*p* = 0.091), hypercholesterolemia (*p* = 0.807), physical inactivity (*p* = 0.621), and heart disease (*p* = 0.979).

Participants without assessment of WMH at baseline tended to be older (*p* < 0.0001), had higher LIBRA-scores (*p* = 0.0003), worse cognitive performance (*p* < 0.0001) and were more likely to have a low SES (*p* = 0.012), live alone (*p* = 0.013), or be retired (*p* < 0.0001). Missing information on HCV at baseline was linked to older age (*p* < 0.0001), living alone (*p* = 0.042), being retired (*p* < 0.0001), and worse cognitive performance (*p* = 0.002).

Individuals who did not participate in the follow-up examination differed from participants regarding certain LIBRA-factors. Those who did not attend the follow-up examination were more likely to be smokers (*p* < 0.0001), report depressive symptoms (*p* = 0.014), and less likely to consume a healthy diet (*p* < 0.0001), but more likely to be hypertensive (*p* < 0.0001), or report a diagnosis of renal dysfunction (*p* < 0.001) or hypercholesterolemia (*p* = 0.042) than participants.

### Change in cognitive performance

Higher LIBRA-scores at baseline were linked to lower levels of cognitive performance in univariate analyses (composite z-score; b = −0.27; 95% CI: −0.35, −0.20; not tabulated). Changes in cognitive performance are displayed in Table 2. A high, but not an intermediate level of SES was linked to better performance at follow-up, controlling for covariates (b = 0.12; 95% CI: 0.01, 0.24). While changes (delta, Δ) in the continuous (original) LIBRA-score were not linked to cognitive performance at follow-up (b = 0.001, 95% CI: −0.02, 0.02), squared values of ΔLIBRA were linked to worse cognitive performance (b = −0.007, 95% CI: −0.01, −0.001), controlling for covariates (age, sex, employment status, relationship status, living situation, social isolation, social support, time between baseline and follow-up, and baseline cognitive performance). No signs of major collinearity were detected, with all VIFs ≤ 4.43 (mean VIF: 1.96; see Supplementary Table 4). In sensitivity analyses, applying a reduced model with age, sex, SES, ΔLIBRA, ΔLIBRA^2^, baseline cognitive performance and time between examinations as predictors, associations remained unchanged (see Supplementary Table 5). Supplementary analyses, using the same model with individual cognitive tests as outcomes (TMT-A, TMT-B, VFT) revealed highly comparable results. Changes in the continuous LIBRA-score were not associated with performance in individual cognitive tests, while squared values of ΔLIBRA were linked to worse performance in TMT-A (b = −0.01, 95% CI: −0.01, −0.0003) and TMT-B (b = −0.01; 95% CI: −0.02, −0.002). However, associations did not survive the FDR-correction for multiple testing. Intermediate and high levels of SES were linked to better performance in the VFT (b_intermediate_ = 0.14, 95% CI: 0.03, 0.26; b_high_ = 0.27, 95% CI: 0.14, 0.40), while only the latter remained significant after adjusting for multiple testing; see Supplementary Table 6).

**Table 2:** Multivariable regression analyses of cognitive, lifestyle, and neuroimaging outcomes

**Outcome: Cognitive performance (composite z-score at follow-up; *n* = 1,581)**				
**Predictor**	**b**	**95% CI**	**p (unadjusted)**	
Δ LIBRA	0.001	−0.02, 0.02	.911	
Δ LIBRA^2^	−0.007	−0.01, −0.001	.020	
SES intermediate (ref.: low)	0.08	−0.02, 0.17	.113	
SES high	0.12	0.01, 0.24	.028	
**Outcome: Lifestyle (LIBRA-score; *n* = 1,581)**				
**Predictor**	**b**	**95% CI**	**p (unadjusted)**	**p (FDR-adjusted)**
SES intermediate (ref.: low)	−0.09	−0.16, −0.02	.010	.030
SES high	−0.12	−0.20, −0.03	.006	.027
**Outcomes: Neuroimaging markers**				
	**b (1,000 mm**^3^)	**95% CI (1,000 mm**^3^)	**p (unadjusted)**	**p (FDR-adjusted)**
Hippocampal volume (*n* = 902)				
Predictor				
Δ LIBRA	−0.001	−0.007, 0.005	.690	.690
Δ LIBRA^2^	−0.0002	−0.002, 0.002	.830	.830
SES intermediate (ref.: low)	−0.02	−0.05, 0.01	.216	.216
SES high	−0.03	−0.07, 0.004	.084	.084
White matter hyperintensity volume (*n* = 889)				
Predictor				
Δ LIBRA	0.02	0.003, 0.03	.016	.016
Δ LIBRA^2^	−0.001	−0.01, 0.003	.643	.643
SES intermediate (ref.: low)	−0.09	−0.15, −0.02	.017	.017
SES high	−0.02	−0.10, 0.05	.546	.546

*CI* Confidence interval, *FDR* False discovery rate, *LIBRA* Lifestyle for Brain Health Index, *ref.* reference, *SES* Socioeconomic status, *ΔLIBRA* LIBRA (total score) at follow-up—LIBRA (total score) at baseline; all analyses adjusted for age, sex, employment status, relationship status, living situation, social isolation, social support, time between baseline and follow-up, and baseline values of respective outcomes. p-values were adjusted for multiple testing of focal predictors in secondary analyses (LIBRA-FU, white matter hyperintensity volume, hippocampal volume) using the Benjamini–Hochberg false discovery rate (FDR). Primary outcome analyses (cognitive performance, z-score) reported with unadjusted *p*-values. Volumetric MRI outcomes described in units of 1,000 mm^3^

To test whether the association between lifestyle changes (ΔLIBRA^2^) and cognitive performance differed by SES group, an additional model including an interaction term (ΔLIBRA^2^*SES) was calculated. A pooled Wald test using Rubin’s rules indicated no significant interaction (F(2, 527.7) = 0.33; *p* = 0.718). Similar results were observed when testing an interaction between LIBRA at baseline and SES (results not shown). Based on this finding, no SES-stratified models or interaction terms were retained (not tabulated). Further sensitivity analyses were conducted, adding baseline values of HCV and WMH volume as predictors of cognitive performance at follow-up. Higher HCV was linked to better cognitive performance at follow-up (b = 178.16; 95% CI: 77.43, 278.89), while higher WHH volumes were associated with worse cognitive performance (b = −0.05; 95% CI: −0.09, −0.01). Associations of ΔLIBRA and ΔLIBRA^2^ with cognitive performance remained unchanged (*p* = 0.400 and *p* = 0.015, respectively; not tabulated). Additionally, we assessed whether associations of HCV and WMH volumes with cognitive performance were moderated by LIBRA-tertiles (i.e., lower, intermediate, higher lifestyle-based risk for dementia). No significant interactions of HCV and WMH volumes with LIBRA-tertiles were detected (all *p*-values ≥ 0.265, respectively), and inclusion of interaction terms rendered associations of HCV and WMH volumes with cognitive performance non-significant (see Supplementary Table 7).

**Fig. 1:**
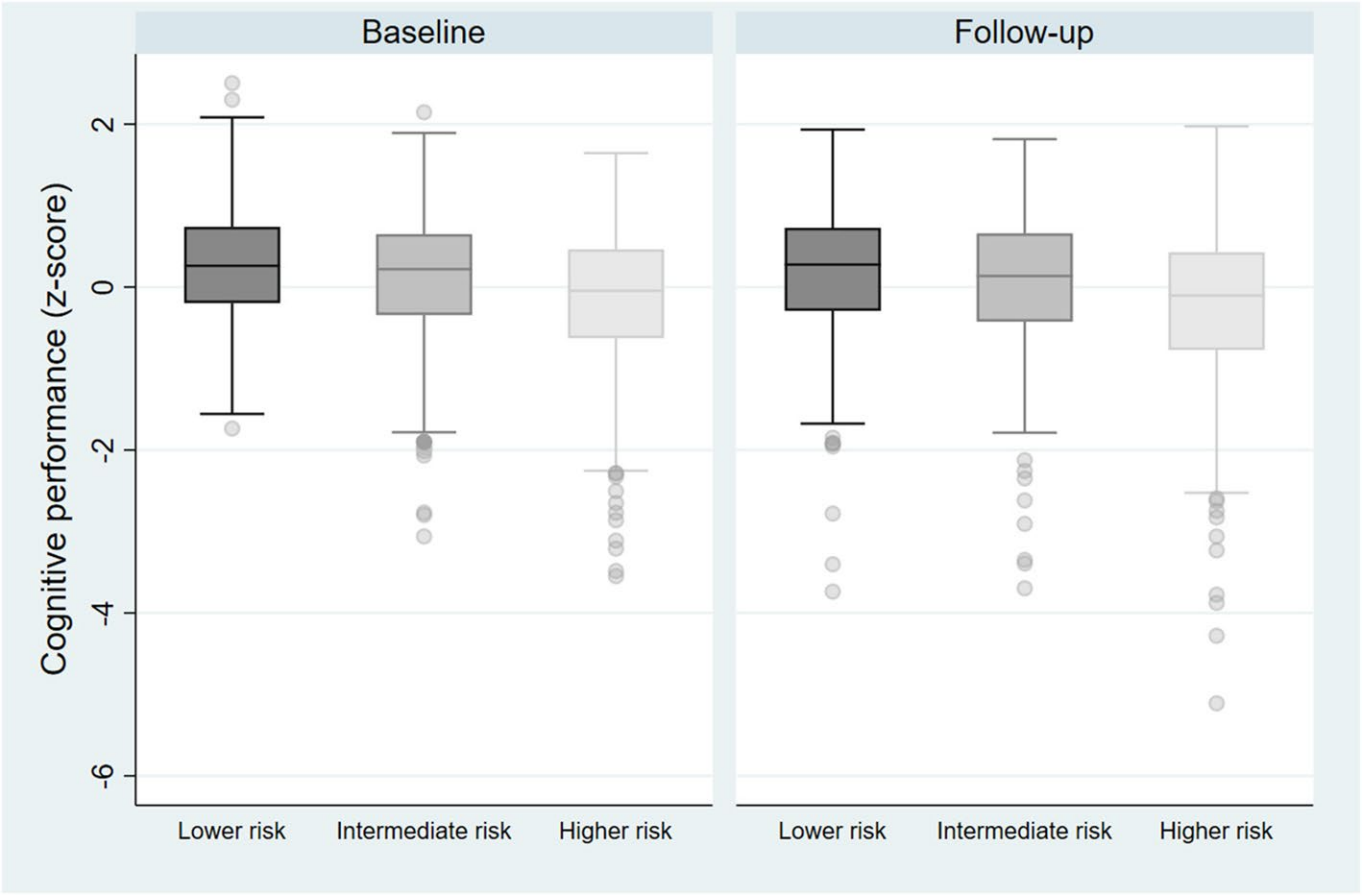
Cognitive performance, by LIBRA-tertiles. Cognitive performance (composite z-score) at baseline and follow-up, by LIBRA-tertiles

**Fig. 2:**
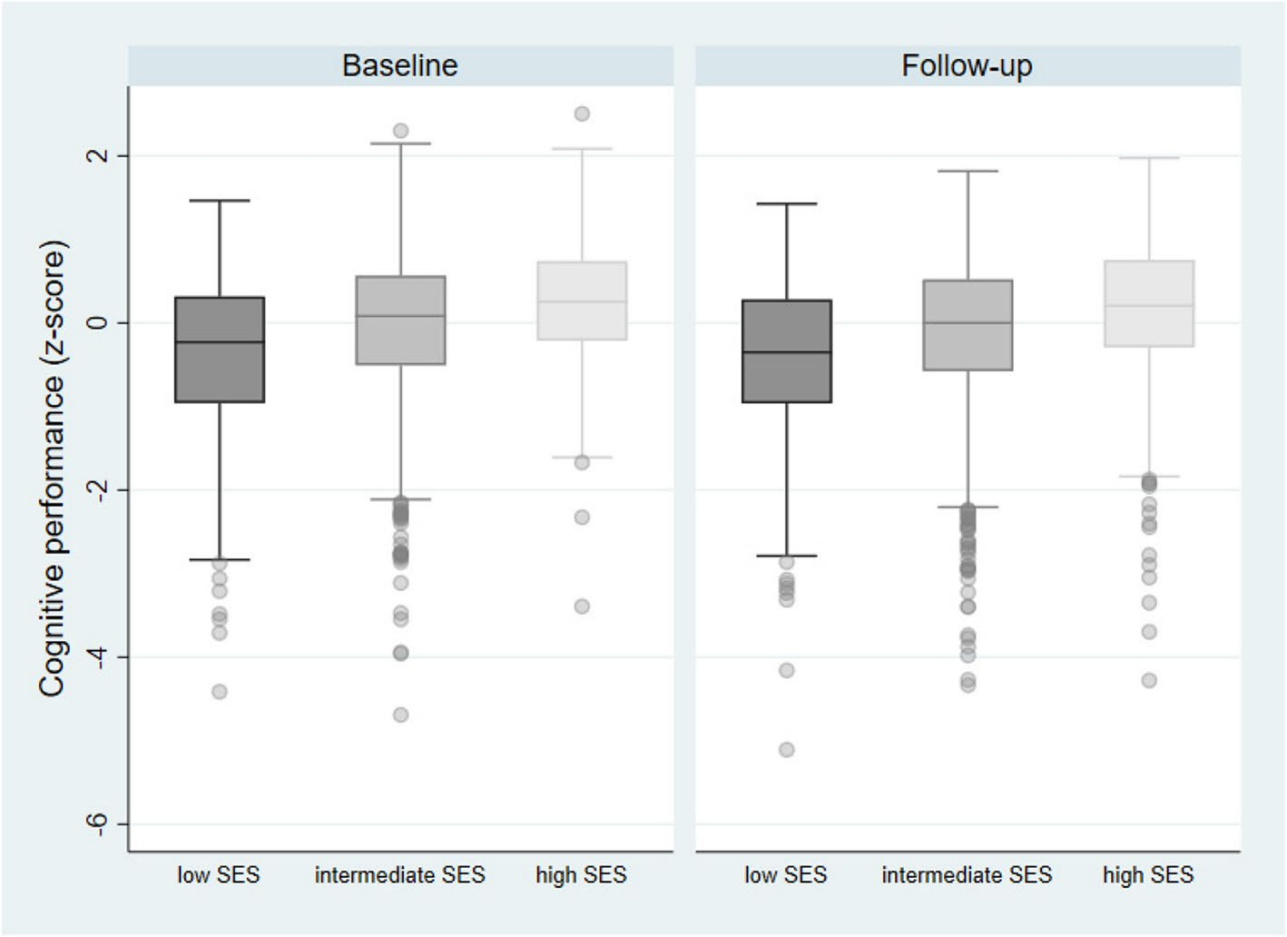
Cognitive performance, by SES-groups. Cognitive performance (composite z-score) at baseline and follow-up, by SES; SES: socioeconomic status

Cognitive performance by LIBRA-tertile (lower, intermediate, higher risk) at baseline and follow-up is displayed in Fig. 1. Figure 2 displays cognitive performance at baseline and follow-up by SES groups.

To assess robustness of our findings regarding changes in cognitive performance, we conducted sensitivity analyses using the CERAD “word list learning”-test, assessing memory performance, as outcome. Results were largely comparable, with no significant effect of ΔLIBRA (b = 0.71, 95% CI: −0.02, 0.16) or ΔLIBRA^2^ (b = −0.01; 95% CI: −0.05, 0.02). A high SES was linked to better performance in the CERAD word list learning-test (b = 0.66; 95% CI: 0.11, 1.21).

### Change in dementia risk (LIBRA)

On average, LIBRA increased across all SES-groups in univariate analyses (low SES: mean ΔLIBRA = 0.64 points; 95% CI: 0.36, 0.91; intermediate SES: mean ΔLIBRA = 0.28 points; 95% CI: 0.15, 0.41; high SES: mean ΔLIBRA = 0.35 points; 95% CI: 0.14, 0.56), indicating higher modifiable dementia risk at follow-up. While greater changes were observed in the lower SES-group in univariate analyses, these differences were not significant, as indicated by Wald tests (F(2, 860) = 2.80, *p* = 0.061; not tabulated). In multivariable regression analyses, an intermediate (b = −0.09; 95% CI: −0.16, −0.02) and high SES at baseline (b = −0.12; 95% CI: −0.20, −0.03) was linked to lower LIBRA-scores at follow-up, indicating a lower risk for dementia (Table 2). Exploratory analyses assessed changes in LIBRA, using baseline tertiles of LIBRA as a predictor (low/moderate/high risk), controlling for covariates as described above, including baseline cognitive performance. Individuals with moderate (b = −0.99; 95% CI: −1.23, −0.76) and high (b = −2.11; 95% CI: −2–36, −1.87) risk at baseline exhibited greater reduction of LIBRA at follow-up. Individuals within the intermediate (b = −0.40, 95% CI: −0.67, −0.12) and high SES-groups (b = −0.42, 95% CI: −0.75, −0.10) showed reductions in LIBRA. Interaction analyses (age × LIBRA-tertile, sex × LIBRA-tertile, SES × LIBRA-tertile) did not indicate effect moderation (all *p* > 0.18; not tabulated).

### Change in neuroimaging markers

At baseline, higher LIBRA-scores were associated with higher WMH volumes (b = 0.14; 95% CI: 0.03, 0.45; *p* = 0.012, not tabulated), controlling for covariates as described above. No significant association of LIBRA with HCV at baseline was detected when controlling for covariates (b = −0.0006; 95% CI: −0.02, 0.004; *p* = 0.244; not tabulated).

At follow-up, higher WMH volume was linked to unfavorable changes in lifestyle (ΔLIBRA) when adjusting for covariates, including baseline WMH volume (b = 0.02; 95% CI: 0.003, 03). An intermediate level of SES was linked to lower WMH volume at follow-up (b = 0.09; 95% CI: −0.15; −0.02). No effect of ΔLIBRA was detected on HCV at follow-up after controlling for covariates (b = − 0.001; 95% CI: − 0.007, 0.005; Table 2). Sensitivity analyses, using a combined MRI-dataset including participants with both WMH volumes and HCV at baseline and follow-up (*n* = 875) revealed highly comparable results (Supplementary Table 8). Graphic descriptions of multivariable regression analyses investigating changes in cognitive performance, LIBRA, and neuroimaging markers are provided in the Supplementary Figs. 1–5.

## Discussion

### Change in cognitive performance

In the present study from a registry-based sample of middle-aged and older German adults, low SES predicted negative changes in cognitive performance. Lifestyle changes, assessed using a validated dementia risk score (LIBRA), were linked to changes in cognition only beyond a certain threshold, suggesting that only changes in LIBRA beyond a certain level predict decreases in cognitive performance. This pattern may reflect threshold effects, whereby only substantial cumulative increases in modifiable risk factors—potentially indicating broader health deterioration—translate into changes in cognitive performance. Significant changes in health behavior (regardless of direction) might reflect underlying vulnerability or instability in health status not captured by the variables included in our analyses. For example, individuals who improve their LIBRA-profile may do so in response to prior health shocks or medical diagnoses (e.g., stroke), which themselves are associated with accelerated cognitive decline. Conversely, individuals whose LIBRA-profiles worsen may be experiencing emerging or worsening health issues that directly impact brain health. Another possible explanation refers to rather small changes in LIBRA-scores (mean change in the total sample: 0.34 points). Participation in the LIFE-Adult-Study was, on average, linked to healthier lifestyle and lower burden of disease, limiting generalizability to the wider adult population in Germany [30]. On another note, our analyses included a large number of covariates, controlling for potential influences of baseline cognitive performance, as well as sociodemographic and psychosocial determinants of health, which may explain the non-significant linear association between LIBRA and changes in cognition in our sample. Average time between baseline- and follow-up examination was 6.6 years, which might be too short to capture lifestyle-related changes on cognitive performance in a generally healthy, registry-based sample of middle-aged and older adults.

Certain studies suggest that including further factors into risk scores like LIBRA, e.g., history of concussion [53], might increase predictive performance for cognitive decline and dementia. A recent update of the LIBRA-index further included sleep problems, hearing impairment and social contact [54], however, information on these factors was limited in the LIFE-Adult-Study, therefore, we applied the original version of the risk score. Further dimensions of socioeconomic differences, e.g., measures of area-level deprivation like neighborhood safety or (lack of) access to green spaces, have been suggested as promising factors to explain differences in cognitive performance/decline [55]. Future longitudinal studies including respective measures to increase explanatory power are encouraged.

The significant effect of SES on cognitive performance at follow-up underlines the importance of socioeconomic determinants of health and, to a lesser extent, lifestyle factors, for cognitive performance and brain health in middle-aged and older adults. Higher SES commonly entails better access to resources and opportunities for a brain-healthy lifestyle, but also shapes exposure to risk and protective factors for cognitive decline and dementia, e.g., people with a high SES are less likely to live and work in areas with greater exposure to environmental risk factors [56]. Lower SES is also closely linked to individual and area-level deprivation, including factors like job stability or neighborhood safety, which further contribute to risk of cognitive decline and dementia [57], as well as greater exposure to psychosocial stressors which increase dementia risk [58].

Our findings revealed no indication for an interaction between SES and lifestyle, suggesting similar associations of LIBRA and changes in cognition across SES-groups. These results are in line with findings from two Dutch cohort studies [59, 60], the UK Biobank [15], and the US-American National Health and Nutrition Examination Survey [61], reporting independent effects of SES ([60]: education) and lifestyle, but no respective interaction effects on cognitive decline and incident dementia. In line with this finding, in the Finnish Geriatric Intervention Study to Prevent Cognitive Decline and Disability (FINGER), effects of the multidomain lifestyle intervention were observed across SES-groups, with no indication of interaction effects [62]. Certain studies found differential effects of healthy lifestyles on cognitive performance, with more pronounced benefits in socially disadvantaged persons in some studies [18], while others reported stronger associations among high-SES individuals [20].

While respective studies from Low and Middle-Income Countries (LMICs) or the Global South simultaneously investigating the role of SES and lifestyle in cognitive performance and dementia are currently limited, results from the Chinese Longitudinal Healthy Longevity Survey suggested that the protective effects of healthy lifestyles against cognitive impairment were stronger among participants with a low SES [63]. Irfani and colleagues reported independent associations of both SES (lower education and lower occupational class) and lifestyle factors and comorbidities (stroke, traumatic brain injury, uncontrolled diabetes, hearing loss, and chronic obstructive airway disease) with dementia in a large sample of adults aged ≥ 65 from Indonesia, however, without assessing potential interaction effects [64]. In another study assessing the prevalence of cognitive impairment and dementia in a multi-ethnic Indigenous sample from Brazil, neither lifestyle and comorbidities nor poverty (as an indicator of SES) were linked to cognitive impairment or dementia in multivariable analyses [65]. Yaffe and colleagues [10] reported elevated risk for incident dementia among Black, compared to White participants in a clinic-based cohort from the US. While lifestyle factors (physical activity, alcohol consumption, smoking, BMI) and comorbidities did not significantly alter the hazard ratio for race, differences in incident dementia between White and Black participants were no longer significant when controlling for SES.

### Change in dementia risk (LIBRA)

Regarding changes in modifiable risk factors, our findings corroborate the well-established findings describing a socioeconomic gradient in risk factors for dementia: Even when controlling for baseline-LIBRA and a large set of covariates, individuals with a low SES had higher LIBRA-scores at follow-up, compared to individuals with a high SES. This points towards the need for addressing inequalities in (brain-)healthy ageing across social strata, making the case for (population-level) approaches that facilitate healthy lifestyle choices for all [12] and rely less on individual choices and behavior. Even in a high-income country like Germany, the risk of coronary heart disease, diabetes mellitus, chronic obstructive pulmonary disease, and depression is two to three times higher among people with a low SES than among those from the high SES-group [66], and gaps in life expectancy between the most and least deprived areas increased from 2.6 (women) and 5.7 (men) years to 4.3 (women) and 7.2 (men) years in the last two decades [67]. Calls for addressing material, psychosocial and structural barriers for brain-healthy ageing are increasingly being made [68–70]: First of all, because individual resources and opportunities for healthy lifestyle choices are distributed unequally within societies; secondly, certain potentially modifiable risk/protective factors for dementia are not amendable by individual choice, e.g., exposure to air pollution, pesticides or neighborhood characteristics such as green spaces [71–73]. Future studies including a broader range of socioeconomic and environmental factors which might contribute to lifestyle in older age are encouraged to improve our understanding on what shapes modifiable dementia risk in middle-aged and older adults.

### Change in neuroimaging markers

We found evidence that higher LIBRA-scores were linked to greater WMH cross-sectionally, while no association with HCV was detected when controlling for covariates (sociodemographic, psychosocial, and cognitive factors). This might indicate that unhealthier lifestyles are linked to markers of cerebral vascular damage, but less so to indicators of hippocampal atrophy in our sample. Associations of LIBRA with WMH/cerebral small vessel disease burden were also reported in population-based studies from the Netherlands [25] and China [21]. LIBRA comprises several risk factors for cerebral vascular damage (e.g., diabetes, physical inactivity, smoking, hypertension), which can negatively impact endothelial function, microangiopathy and markers of inflammation. This might explain the observed association between changes in LIBRA and higher WMH volume at follow-up, corroborating findings from previous studies [24–27]. Evidence from intervention studies further suggest greater immediate responses of WMH volume to lifestyle interventions than HCV [74]. On another note, indirect effects of WMH volume on hippocampal volumes and cognitive performance over time have been suggested, whereas effects of lifestyle changes are observed more immediate in WMH volumes than in long-term structural changes indicated by hippocampal atrophy [75]. Our results suggest that a compound score reflecting modifiable dementia risk factors is a suitable tool to detect changes in markers of cerebral small vessel disease and stress the potential for brain-healthy lifestyle changes to attenuate WMH progression. After controlling for baseline values of respective neuroimaging markers and further covariates, no longitudinal association with LIBRA was detected for HCV, an area particularly vulnerable for AD-related atrophy. In line with our negative findings regarding effects of lifestyle on HCV, Rodriguez and colleagues [76] found no association between high mental demands at work, a further protective factors against cognitive decline, and HCV in participants of the LIFE-Adult-Study. Possibly, respective associations might be observable among clinical samples revealing more signs of neurodegeneration typically observed in dementia and AD. As described above, participants with available MRI-data in our study were, on average, younger, had lower LIBRA-scores, worse cognitive performance and more often belonged to higher SES-groups, indicating possible selection bias, which may have resulted in reduced statistical power to detect effects of lifestyle on HCV. Our findings suggest that lifestyle changes are more closely related to vascular brain pathology than to hippocampal atrophy, which may reflect more slowly evolving neurodegenerative processes. Longitudinal studies assessing neuroimaging markers in more diverse samples regarding cognitive performance and SES are encouraged to enhance our understanding of the impact of SES and lifestyle on structural brain health markers.

### Strengths and limitations

Strengths of our study include a large, registry-based sample, with detailed information on numerous sociodemographic and psychosocial covariates, allowing for in-depth investigations of changes in cognitive performance and the role of SES and lifestyle risk factors for dementia. Except for the factor “high cognitive activity”, information on 11 risk/protective factors comprised by the LIBRA-score were available both at baseline and follow-up, and missing data were handled appropriately using imputation techniques to increase statistical power and ensure robustness of our findings. Further, we provide evidence on corresponding changes in neuroimaging markers in a large subsample of participants, contributing to our knowledge on potential pathways underlying associations between changes in lifestyle and cognitive performance.

Limitations arise from a rather low response rate in the registry-based LIFE-Adult Study (31% at baseline) and a high amount of loss to follow-up, particularly among individuals with a low SES, as well as an overrepresentation of individuals with a high SES in the study, compared to the city of Leipzig and non-participants. This may have introduced selection bias, and estimates regarding the impact of SES and lifestyle on cognitive performance might be underestimated in our study. The sample included comparatively educated (84.5% having an intermediate or high level of education [34]), mostly German-born adults from a geographically narrowly defined area, which limits the generalizability of our findings. This limited variability might have contributed to the non-significant interaction between LIBRA and SES. More studies investigating the (joint) contribution of socioeconomic and lifestyle factors for brain health and cognition in large, diverse samples, particularly from the Global South or focusing on populations at increased risk for deprivation, are needed to design effective strategies for dementia risk reduction on a population level and reduce inequalities in healthy ageing. Changes in LIBRA-score between baseline and follow-up were rather small, limiting explanatory power of our analyses. This might, in part, be due to selective participation in LIFE-Adult, with participation linked to higher SES, healthier lifestyle and lower burden of disease than in the general German population or the city of Leipzig, as described earlier [30, 34]. Exploratory analyses indicated greater lifestyle changes in individuals with moderate and high LIBRA at baseline, suggesting ceiling effects, with no indication of modification by sex, age or SES. Due to restrictions related to the COVID19-pandemic, duration between baseline- and follow-up examination differed between participants, however, this was accounted for in multivariable analyses. Since individual factors of the LIBRA are assessed in a binary fashion (yes/no), the index might have missed small changes in lifestyle that may have occurred between baseline and follow-up. Further, some factors comprised by LIBRA (e.g., chronic kidney disease) might be less amendable to change than others (e.g., physical inactivity, diet) within the studied timeframe. Still, LIBRA was highly responsive to lifestyle changes in a study comparing different dementia risk scores [77], underlining its suitability for measuring modifiable dementia risk.

## Conclusion

Our findings provide further evidence for the influence of socioeconomic differences on cognitive performance in middle-aged and older adults. Lifestyle factors, however, explained differences in cognitive performance at follow-up only beyond a certain threshold. Results indicated no interaction between SES and lifestyle (assessed using the LIBRA-score) regarding effects on cognitive performance. On the one hand, these findings indicate that a brain-healthy lifestyle is beneficial for everyone, regardless of SES. On the other, our study also revealed detrimental lifestyle changes in low-SES individuals, which may lead to higher rates of cognitive decline and dementia in the long run. Low-SES groups have a higher prevalence of modifiable risk factors for dementia, arguing for policies that reduce exposure to said risk factors. This might particularly benefit individuals with a low SES and thereby reduce health inequities in (brain)healthy ageing. Lastly, while no effects of LIBRA on HCV were detected in our study, our findings suggest that detrimental lifestyle changes were linked to increases in WMH volume, underlining the potential of dementia risk scores to capture changes in markers of cerebral small vessel disease. Against this background, dementia risk reduction should prioritize populations facing socioeconomic disadvantage, addressing both lifestyle risk factors and broader structural determinants of health in order to mitigate rather than amplify brain health inequities.

## Supplementary Information

Supplementary Material 1.

## Data Availability

Due to privacy protection, restrictions apply to the availability of the data. Data from the LIFE-Adult-Study are available to researchers who submit a detailed written proposal, including objectives, measures, names of all researchers involved, and how results and newly generated data will be returned for further use. Data are provided upon approval by the data use- and access-committee. Inquiries are to be submitted to: info-life@lists.uni-leipzig.de.

## Abbreviations

AD: Alzheimer’s disease
ADNI: Alzheimer's Disease Neuroimaging Initiative
CASMIN: Comparative Analysis of Social Mobility in Industrial Nations
CERAD: Consortium to Establish a Registry for Alzheimer’s Disease
CI: Confidence interval
CSF: Cerebrospinal fluid
ESSI: ENRICHD social support inventory
FDR: False discovery rate
GM: Grey matter
HCV: Hippocampal volume
ICV: Intracranial volume
ISEI: International Socio-Economic-Index of Occupational Status
LIBRA: Lifestyle for Brain Health
LSNS: Lubben Social Network Scale
MRI: Magnetic resonance imaging
Ref.: Reference
SD: Standard deviation
SES: Socioeconomic status
TMT: Trail Making Test
VFT: Verbal Fluency Test
VIF: Variance inflation factor
WMH: White matter hyperintensities
