# Supplementary material for: The impact of socioeconomic status and lifestyle on cognitive aging and brain health: results from the LIFE-Adult-Study
Source: Alzheimers Res Ther. 2026 Mar 24;18:80. doi: 10.1186/s13195-026-02017-4 (PMC13063888; doi:10.1186/s13195-026-02017-4)
Supplement: Supplementary file 1 — Supplementary Material 1. [file 13195_2026_2017_MOESM1_ESM.docx]

**Supplementary material**

**Supplementary Table 1: Operationalization of LIBRA-factors in LIFE Adult**

|  | **Operationalization / Assessment** | **Points** |
| --- | --- | --- |
| **Blood pressure** | SBP <140 mmHg and DBP <90 mmHg and no diagnosis of hypertension | **0** |
|  | SBP ≥140 and/or DBP ≥90 and/or diagnosed hypertension | **1.6** |
| **Cholesterol** | Total cholesterol < 5.2 mmol/l or LDL < 4.2 mmol/l | **0** |
|  | Total cholesterol ≥ 5.2 mmol/l and LDL ≥ 4.2 mmol/l | **1.4** |
| **Obesity** | BMI <30; BMI based on weight and height measurement, assessed using electronic scales (SECA 701, Seca Gmbh & Co KG, precision of 0.01 kg; height was assessed using a stadiometer (SECA 240, precision: 0.1 cm) | **0** |
|  | BMI ≥ 30 | **1.6** |
| **Physical activity** | Self-reported physical activity, measured with the IPAQ-SF level “moderate” or “high”; high: vigorous intensity activity on ≥3 days/week, or any combination of walking, moderate, or vigorous activity on 7 days/week; moderate: ≥5 days/week of walking, moderate, or vigorous activity, or ≥3 days/week of vigorous activity for ≥20 minutes per day, or ≥5 days/week of moderate activity or walking for ≥30 minutes per day | **0** |
|  | IPAQ level “low”; does not meet the criteria for moderate or high activity | **1.1** |
| **Diabetes** | No self-reported diagnosis of diabetes mellitus and HbA1c < 6.5% | **0** |
|  | Self-reported diagnosis of diabetes mellitus or HbA1c ≥ 6.5% | **1.3** |
| **Renal dysfunction** | eGFR^a^ ≥ 60 ml/min per 1.73m and no history of renal dysfunction | **0** |
|  | eGFR ^a^ < 60 ml/min per 1.73m or self-reported diagnosis of renal dysfunction | **1.1** |
| **Coronary heart disease** | No history of angina pectoris/coronary heart disease, heart failure, myocardial infarction, cardiac arrhythmia or tachycardia | **0** |
|  | Self-reported history of angina pectoris/coronary heart disease, heart failure, myocardial infarction, cardiac arrhythmia or tachycardia | **1.0** |
| **Depression** | CES-D score < 16 and/or self-reported diagnosis of depression | **0** |
|  | CES-D score ≥ 16 and/or self-reported diagnosis of depression | **2.1** |
| **Smoking** | Not current smoker (standardized self-report questionnaire) | **0** |
|  | Current smoker (standardized self-report questionnaire) | **1.5** |
| **Alcohol** | Consumed grams of alcohol per day, based on standardized self-report questionnaires; low-to-moderate drinking ≤ to 12g/day (women), ≤ 24g/day (men)^b^ | **-1** |
|  | Consumed grams of alcohol per day >12 g/day (women), > 24g/day (men) | **0** |
| **Healthy diet** | Self-reported consumption of raw, frozen or tinned vegetables or fresh fruit “several times per day”, as assessed by standardized food frequency questionnaire | **-1.7** |
|  | Self-reported consumption of raw, frozen or tinned vegetables or fresh fruit less than “several times per day”, as assessed by standardized FFQ | **0** |

BMI: body mass index; CES-D: Center for Epidemiological Studies Depression Scale; DBP: diastolic blood pressure; eGFR: Estimated glomerular filtration rate; FFQ: food frequency questionnaire; IPAQ: International Physical Activity Questionnaire; LDL: low-density lipoprotein; LIBRA: lifestyle for brain health; IPAQ-SF: short form of the International Physical Activity Questionnaire; SBP: systolic blood pressure

^a^: eGFR calculated using the Stata-package “egfr” (Phil Clayton, 2013: "EGFR: Stata module to calculate estimated glomerular filtration rate (eGFR)," Statistical Software Components S457731, Boston College Department of Economics, revised 04 Aug 2021), applying the Chronic Kidney Disease Epidemiology Collaboration (CKD-EPI) equation for eGFR.

^b^ recommendations of the German *Centre for Addiction Issues (DHS);* <https://www.dhs.de/fileadmin/user_upload/WK_der_DHS_-_Empfehlungen_zum_Umgang_mit_Alkohol.pdf>

**Supplementary Table 2: Associations of item-missingness of imputed variables with observable participant characteristics**

|  | **n missing (%)** | **Age *(p*)** | **Female sex (*p*)** | **SES intermediate (*p*)#** | **SES low (*p*)#** |
| --- | --- | --- | --- | --- | --- |
| Employment status | 4 (0.3) | .479 | .870 | .876 | / |
| Living situation | 2 (0.1) | .826 | .908 | / | / |
| Social isolation | 175 (11.1) | **.002** | **<.001** | .965 | .159 |
| Perceived social support | 34 (2.2) | **.037** | **.010** | .232 | **.019** |
| Cognitive performance (z-score) | 14 (1.0) | .941 | .191 | .854 | .968 |
| *LIBRA-factors* |  |  |  |  |  |
| Blood pressure | 55 (3.5) | .762 | **.016** | .073 | .055 |
| Cholesterol | 10 (0.6) | .946 | .370 | .689 | .568 |
| Obesity | 1 (0.06) | / | .357 | / | / |
| Physical activity | 237 (15.0) | **<.0001** | **.002** | **<.0001** | **<.0001** |
| Diabetes | 14 (1.0) | .469 | .758 | .854 | .968 |
| Renal dysfunction | 8 (0.5) | .650 | .098 | .689 | .754 |
| Coronary heart disease | 20 (1.3) | .794 | .934 | .968 | .924 |
| Depression | 6 (0.4) | .446 | .307 | .294 | .393 |
| Smoking | 58 (3.7) | **.0007** | **.025** | **.018** | **<.0001** |
| Alcohol | 104 (6.6) | **.0002** | .721 | **.028** | **<.001** |
| Healthy diet | 29 (1.8) | **.0004** | .313 | .191 | **.034** |
| LIBRA-score, total | 386 (24.4) | **<.0001** | **<.0001** | **<.0001** | **<.0001** |

Variables age, sex, SES, relationship status and country of birth had no missing values at baseline. MRI imaging markers were not imputed. #: reference: high SES.

**Supplementary Table 3: Participant characteristics at baseline, stratified by SES-group (n = 1,581)**

| **Variable** | **Total (n = 1,581)** | **Low SES (n = 245)** | **Intermediate SES (n = 955)** | **High SES (n = 381)** | ***p*** |
| --- | --- | --- | --- | --- | --- |
| Age, Mean (SD); range | 63.6 (10.1); 40.1 – 80.3 | 64.7 (8.9);  42.4 – 79.1 | 63.8 (10.3); 40.7 – 79.6 | 62.5 (10.3); 40.1 – 80.3 | **.034** |
| Women | 726 (45.9) | 125 (51.0) | 458 (48.0) | 143 (37.5) | **.001** |
| *Employment status* |  |  |  |  |  |
| employed | 619 (39.3) | 65 (26.5) | 355 (37.3) | 199 (52.4) | **<.001** |
| retired | 913 (57.9) | 156 (63.7) | 580 (60.9) | 177 (46.6) |  |
| unemployed, homemaker | 45 (2.9) | 24 (9.8) | 17 (1.8) | 4 (1.1) |  |
| *Country of birth* |  |  |  |  |  |
| Germany | 1,493 (94.4) | 233 (95.1) | 902 (94.5) | 358 (94.0) | .831 |
| other | 88 (5.6) | 12 (4.9) | 53 (5.6) | 23 (6.0) |  |
| *Living situation* |  |  |  |  |  |
| living alone | 312 (19.8) | 91 (37.1) | 165 (17.3) | 56 (14.7) | **<.001** |
| living together with others | 1,267 (80.2) | 154 (62.9) | 788 (82.7) | 325 (85.3) |  |
| *Relationship status* |  |  |  |  |  |
| in a relationship | 1,284 (81.2) | 157 (64.1) | 801 (83.9) | 326 (85.6) | **<.001** |
| single, divorced, widowed | 297 (18.8) | 88 (35.9) | 154 (16.1) | 55 (14.4) |  |
| *Social isolation* |  |  |  |  |  |
| socially isolated (LSNS < 12) | 197 (14.0) | 54 (25.0) | 105 (12.5) | 38 (10.9) | **<.001** |
| socially integrated (LSNS ≥ 12) | 1,209 (86.0) | 162 (75.0) | 736 (87.5) | 311 (89.1) |  |
| Perceived social support (ESSI), Mean (SD) | 22.3 (3.5) | 20.8 (4.7) | 22.5 (3.1) | 22.8 (3.1) | **<.001** |
| LIBRA-Score (total), Mean (SD) | 1.5 (2.1) | 1.7 (2.2) | 1.6 (2.2) | 1.2 (2.1) | **.013** |
| Cognitive performance (z-score), Mean (SD) | 0 (0.9) | -0.4 (1.0) | 0 (0.9) | 0.2 (0.8) | **.0001** |
| Hippocampal volume (1,000m³), Mean (SD) | 8.3 (0.9) | 8.0 (0.9) | 8.3 (0.9) | 8.4 (0.8) | **.0001** |
| Volume of right hippocampus (1,000m³), Mean (SD) | 4.2 (0.4) | 4.1 (0.4) | 4.2 (0.5) | 4.3 (0.4) | **.0007** |
| Volume of left hippocampus (1,000m³), Mean (SD) | 4.1 (0.4) | 4.0 (0.4) | 4.1 (0.4) | 4.2 (0.4) | **.0004** |
| White matter hyperintensity volume (1,000m³), Mean (SD) | 2.4 (5.9) | 2.0 (3.8) | 2.6 (6.5) | 2.4 (5.6) | .995 |
| Total intracranial volume (1,000m^3^), Mean (SD) | 1,572.0 (142.4) | 1,527.4 (124.7) | 1,570.6 (142.0) | 1,600.2 (146.5) | **.0001** |

ESSI: ENRICHD social support inventory; LIBRA: Lifestyle for Brain Health; LSNS: Lubben Social Network Scale; ref.: reference; SD: standard deviation

**Supplementary Table 4: Variance inflation factors** **for multivariable regression analysis of changes in cognitive performance**

| **Predictor** | **VIF** | **1/VIF** |
| --- | --- | --- |
| Age | 2.71 | 0.37 |
| Female sex (ref.: male) | 1.08 | 0.93 |
| SES intermediate (ref.: low) | 2.16 | 0.46 |
| SES high | 2.24 | 0.45 |
| Cognitive performance at baseline | 1.29 | 0.77 |
| Δ LIBRA | 1.06 | 0.95 |
| Δ LIBRA² | 1.06 | 0.95 |
| Living alone (ref.: living together with others) | 4.39 | 0.23 |
| In a relationship (ref.: single / divorced / widowed) | 4.43 | 0.23 |
| Socially isolated (ref.: socially integrated) | 1.13 | 0.88 |
| Perceived social support | 1.22 | 0.82 |
| Time between baseline and follow-up examination | 1.10 | 0.91 |
| **Mean** | 1.96 |  |

LIBRA: Lifestyle for Brain Health; ref.: reference; VIF: variance inflation factor

**Supplementary Table 5: Multivariable regression analyses of cognitive, lifestyle, and neuroimaging outcomes, reduced set of covariates**

| **Outcome: Cognitive performance (composite z-score at follow-up; n = 1,581)** | | | |
| --- | --- | --- | --- |
| **Predictor** | **b** | **95% CI** | **p (unadjusted)** |
| Δ LIBRA | 0.0007 | -0.02, 0.02 | .942 |
| Δ LIBRA² | -0.007 | -0.01, -0.001 | **.018** |
| SES intermediate (ref.: low) | 0.08 | -0.01, 0.17 | .098 |
| SES high | 0.13 | 0.02, 0.24 | **.016** |
| **Outcome: Lifestyle (LIBRA-score; n = 1,581)** | | | |
| **Predictor** | **b** | **95% CI** | **p (unadjusted)** |
| SES intermediate (ref.: low) | -0.11 | -0.18, -0.04 | **.001** |
| SES high | -0.14 | -0.22, -0.06 | **.001** |
| **Outcomes: Neuroimaging markers** | | | |
|  | **b (1,000 mm³)** | **95% CI (1,000 mm³)** | **p (unadjusted)** |
| **Hippocampal volume (n = 902)** |  |  |  |
| **Predictor** |  |  |  |
| Δ LIBRA | -0.001 | -0.007, 0.004 | .646 |
| Δ LIBRA² | -0.000 | -0.002, 0.002 | .834 |
| SES intermediate (ref.: low) | -0.02 | -0.05, 0.01 | .222 |
| SES high | -0.03 | -0.06, 0.005 | .097 |
| **White matter hyperintensity volume (n = 889)** | | | |
| **Predictor** |  |  |  |
| Δ LIBRA | 0.02 | 0.003, 0.03 | **.016** |
| Δ LIBRA² | -0.001 | -0.01, 0.003 | .541 |
| SES intermediate (ref.: low) | -0.08 | -0.14, -0.01 | **.026** |
| SES high | -0.008 | -0.08, 0.07 | .828 |

Notes: CI = confidence interval; LIBRA = Lifestyle for Brain Health Index; ref. = reference; SES: socioeconomic status; ΔLIBRA = LIBRA (total score) at follow-up - LIBRA (total score) at baseline; all analyses adjusted for age, sex, time between baseline and follow-up, and baseline values of respective outcomes. Volumetric MRI outcomes described in units of 1,000 mm³.

**Supplementary Table 6: Multivariable regression analyses of cognitive performance at follow-up, individual cognitive tests**

| **Outcome: Trail Making Test A; n = 1,581** | | | | |
| --- | --- | --- | --- | --- |
| **Predictor** | **b** | **95% CI** | **p (unadjusted)** | **p (FDR-adjusted)** |
| Δ LIBRA | 0.02 | -0.02; 0.04 | .071 | .170 |
| Δ LIBRA² | -0.01 | -0.01; -0.0003 | **.042** | .126 |
| SES intermediate (ref.: low) | 0.05 | -0.06; 0.17 | .363 | .484 |
| SES high | 0.04 | -0.09; 0.18 | .523 | .628 |
| **Outcome: Trail Making Test B; n = 1,581** | | | | |
| **Predictor** | **b** | **95% CI** | **p (unadjusted)** | **p (FDR-adjusted)** |
| Δ LIBRA | 0.001 | -0.02; 0.02 | .908 | .908 |
| Δ LIBRA² | -0.01 | -0.02; -0.002 | **.017** | .068 |
| SES intermediate (ref.: low) | 0.06 | -0.05; 0.17 | .249 | .374 |
| SES high | 0.10 | -0.02; 0.23 | .111 | .222 |
| **Outcome: Verbal Fluency Test; n = 1,581** | | | | |
| **Predictor** | **b** | **95% CI** | **p (unadjusted)** | **p (FDR-adjusted)** |
| Δ LIBRA | -0.01 | -0.04; 0.01 | .181 | .310 |
| Δ LIBRA² | -0.002 | -0.01; 0.01 | .602 | .657 |
| SES intermediate (ref.: low) | 0.14 | 0.03; 0.26 | **.011** | .066 |
| SES high | 0.27 | 0.14; 0.40 | **<.001** | **.001** |

Notes: CI = confidence interval; FDR: false discovery rate; LIBRA = Lifestyle for Brain Health Index; ref. = reference; SES: socioeconomic status; ΔLIBRA = LIBRA (total score) at follow-up - LIBRA (total score) at baseline; all analyses adjusted for age, sex, employment status, relationship status, living situation, social isolation, social support, time between baseline and follow-up, and baseline values of respective outcomes. p-values were adjusted for multiple testing using the Benjamini–Hochberg false discovery rate.

**Supplementary Table 7: Multivariable regression analyses of cognitive performance, including effects of HCV, WMH volumes and interaction with LIBRA**

|  | **b** | **95% CI** | **p** |
| --- | --- | --- | --- |
| **Outcome: Cognitive performance (composite z-score at follow-up)** | | | |
| **Predictor** |  |  |  |
| SES intermediate (ref.: low) | -0.01 | -0.14; 0.11 | .840 |
| SES high | 0.07 | -0.08; 0.21 | .360 |
| HCV at baseline | 152.12 | -2.98; 307.22 | .055 |
| HCV * LIBRA intermediate (ref.: lower) | 33.57 | -174.94; 242.07 | .752 |
| HCV * LIBRA higher (ref.: lower) | 40.25 | -172.34; 252.84 | .710 |
| WMH volume at baseline | -0.03 | -0.10; 0.04 | .399 |
| WMH volume * LIBRA intermediate (ref.: lower) | -0.009 | -0.10; 0.08 | .845 |
| WMH volume * LIBRA higher (ref.: lower) | -0.05 | -0.14; 0.04 | .265 |

Notes: CI = confidence interval; HCV = hippocampal volume; LIBRA = Lifestyle for Brain Health Index; ref. = reference; SES: socioeconomic status; WMH = white matter hyperintensities; LIBRA = LIBRA (total score); analyses adjusted for age, sex, employment status, relationship status, living situation, social isolation, social support, time between baseline and follow-up, and baseline cognitive performance.

**Supplementary Table 8: Multivariable regression analyses of MRI neuroimaging markers at follow-up, combined dataset**

| **Outcomes: Neuroimaging markers** | | | |
| --- | --- | --- | --- |
|  | **b (1,000 mm³)** | **95% CI (1,000 mm³)** | **p** |
| **Hippocampal volume (n = 875)** | | | |
| **Predictor** |  |  |  |
| Δ LIBRA | -0.001 | -0.007, 0.004 | .656 |
| Δ LIBRA² | 0.000 | -0.002, 0.002 | .735 |
| SES intermediate (ref.: low) | -0.02 | -0.05, 0.01 | .256 |
| SES high | -0.02 | -0.06, 0.01 | .204 |
| **White matter hyperintensity volume (n = 875)** | | | |
| **Predictor** |  |  |  |
| Δ LIBRA | 0.02 | 0.003, 0.03 | **.015** |
| Δ LIBRA² | -0.001 | -0.01, 0.003 | .619 |
| SES intermediate (ref.: low) | -0.08 | -0.15, -0.01 | **.024** |
| SES high | -0.02 | -0.10, 0.06 | .631 |

Notes: CI = confidence interval; LIBRA = Lifestyle for Brain Health Index; ref. = reference; SES: socioeconomic status; ΔLIBRA = LIBRA (total score) at follow-up - LIBRA (total score) at baseline; all analyses adjusted for age, sex, employment status, relationship status, living situation, social isolation, social support, time between baseline and follow-up, and baseline values of respective outcomes.

**Supplementary Figure 1: Association between lifestyle change and cognitive performance**


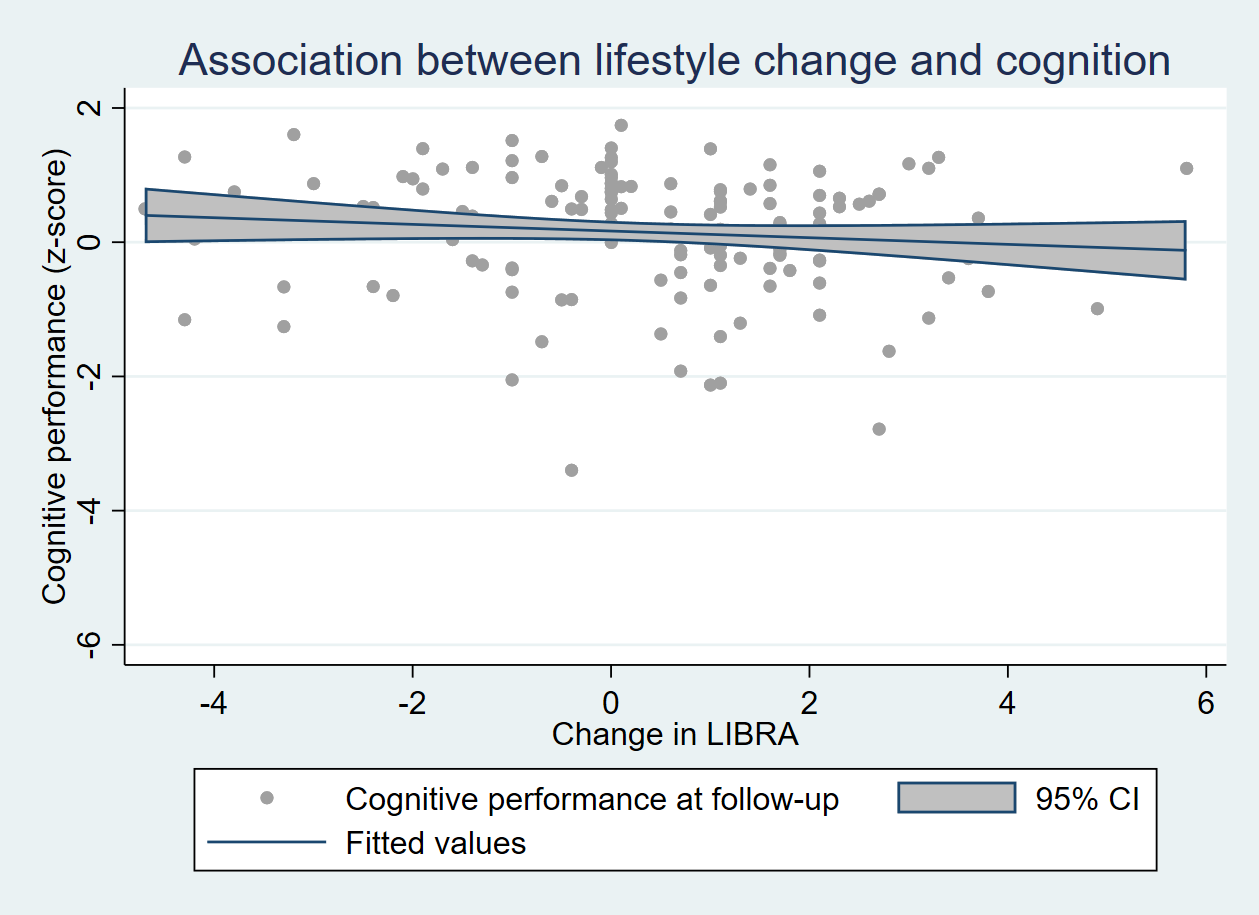


Scatterplot describing the association between change in lifestyle-based dementia risk (ΔLIBRA) and cognitive performance at follow-up. Points represent individual observations. The solid line indicates the fitted linear regression, with the shaded area representing the 95% confidence interval. The plot illustrates the association summarized in the multivariable regression models.

**Supplementary Figure 2: Adjusted change in lifestyle risk by socioeconomic status**


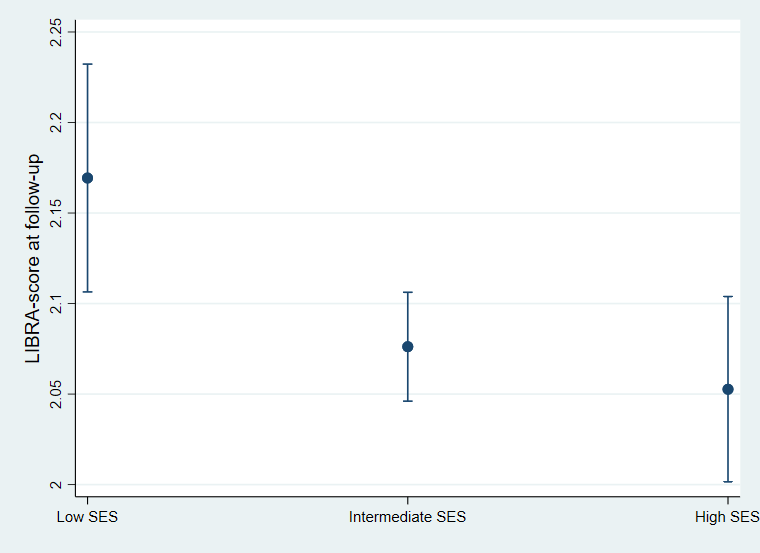


Adjusted mean LIBRA-scores at follow-up by socioeconomic status (SES), estimated from a multivariable regression model including baseline LIBRA and covariates. Points indicate adjusted group means. Error bars represent 95% confidence intervals.

**Supplementary Figure 3: Association between lifestyle change and hippocampal volume**


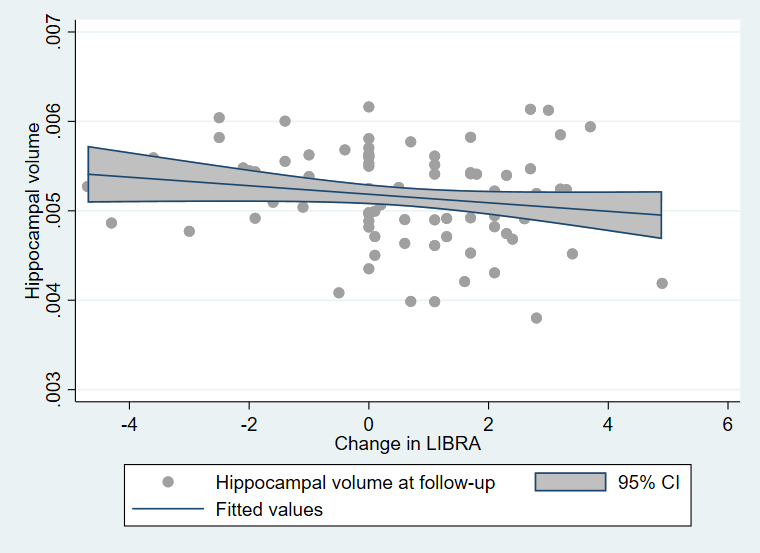


Scatterplot showing the association between change in lifestyle-based risk factors for dementia (ΔLIBRA) and total hippocampal volume at follow-up. Points represent individual observations. The solid line indicates the fitted linear regression with 95% confidence interval. The figure visually describes the association summarized in the multivariable regression analysis.

**Supplementary Figure 4: Association between lifestyle change and white matter hyperintensity volume**


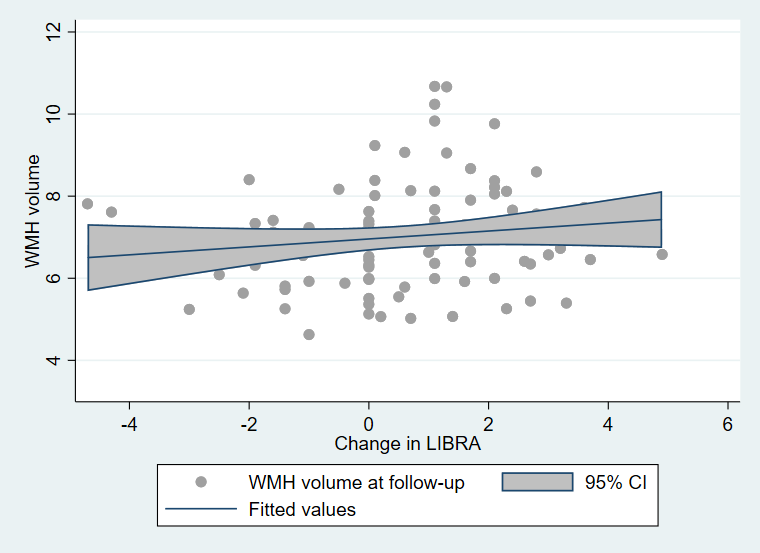


Scatterplot illustrating the association between change in lifestyle-based risk factors for dementia (ΔLIBRA) and white matter hyperintensity (WMH) volume at follow-up. Points represent individual observations. The solid line denotes the fitted linear regression, the shaded area the corresponding 95% confidence interval.
